# Editorial: Molecular mechanisms of sensorineural hearing loss and hearing protection

**DOI:** 10.3389/fnmol.2024.1401219

**Published:** 2024-04-03

**Authors:** Srdjan M. Vlajkovic, A. Catalina Vélez-Ortega, Jennie M. E. Cederholm

**Affiliations:** ^1^Department of Physiology and The Eisdell Moore Centre, Faculty of Medical and Health Sciences, The University of Auckland, Auckland, New Zealand; ^2^Department of Physiology, University of Kentucky, Lexington, KY, United States; ^3^Translational Neuroscience Facility and Department of Physiology, School of Biomedical Sciences, UNSW Sydney, Sydney, NSW, Australia

**Keywords:** hearing loss, hearing protection, blood labyrinth barrier, gap junctions and connexin hemichannels, aminoglycoside ototoxicity, age-related hearing loss (ARHL), sirtuins, cholesterol

More than 1.5 billion people experience declining hearing ability during their lifetime. With an aging population and increased exposure to excessive noise, there has never been a higher demand for understanding the mechanisms of sensorineural hearing loss (SNHL). SNHL arises from congenital and acquired causes, such as age-related cochlear degeneration, acute or chronic exposure to noise, inner ear disease, and ototoxic drugs. Each year, more people are identified as having a genetic mutation linked to SNHL. Bringing together the most current knowledge and advancements in the field of SNHL is vital to the future development of hearing loss treatments. Considerable progress has been made in understanding hearing loss in recent years, and this Research Topic highlighted this progress. In this Research Topic, we present three original and three review articles that add to the current knowledge and advance the field of auditory neuroscience. These articles are briefly summarized here.

Connexin 26 (Cx26) and connexin 30 (Cx30) are prevalent gap junctions in the mammalian cochlea, enabling direct communication between epithelial and connective tissues. Mutations in the genes encoding the Cx26 (*GJB2)* and Cx30 (*GJB6)* are the most common causes of hereditary deafness. Liu and Rask-Andersen analyzed the expression of *GJB2* and *GJB6* transcripts in the human cochlea using the novel RNAscope *in situ* hybridization technique. The *GJB2* and *GJB6* gene transcripts were often co-localized but also showed differential expression in the stria vascularis. Their findings suggest that the gap junction network in the human cochlea contains connexin hemichannels with various permeability and gating characteristics, which may have specific roles in regulating cochlear homeostasis.

Aminoglycoside antibiotics, such as gentamycin or streptomycin, have ototoxic properties that induce the loss of sensory hair cells in the inner ear, resulting in permanent hearing loss. Tao and Segil investigated the role of cyclin-dependent kinase (CDK) activity in aminoglycoside-induced hair cell death using organotypic tissue cultures of the neonatal mouse cochlea. They demonstrated that more hair cells survive gentamycin ototoxicity after disrupting CDK2 activity by pharmacological inhibition or genetic deletion. Their tissue culture and *in vivo* studies suggested CDK2 regulates aminoglycoside-induced apoptosis in sensory hair cells by reducing proapoptotic JNK signaling activity. This study also demonstrated an otoprotective effect of CDK2 inactivation against aminoglycoside ototoxicity in adult mice, revealing a promising target for preventing drug-induced hearing loss.

The blood-labyrinth barrier (BLB) maintains the inner ear fluid ionic homeostasis and is a selective barrier for various substances reaching the inner ear. Impaired BLB integrity has been found in Ménière's disease, acoustic trauma, age-related hearing loss, and autoimmune inner ear disease. The study by Sekulic et al. used a Transwell model to culture human stria vascularis-derived primary endothelial cells (EC) and pericytes on each side of the porous membrane. They exposed the ECs to inflammatory cytokines (TNF-α, IL-6), and lipopolysaccharide (LPS) and observed their influence on endothelial permeability. This enabled them to create an inflammatory environment that affected BLB permeability and modeled inflammatory conditions in the stria vascularis. Designing a functional human BLB model is an excellent step toward understanding normal BLB physiology in humans and testing new therapeutics for inner ear disease in conditions of damaged BLB.

The review by Ohlemiller et al. also focused on BLB. The blood-stria barrier is essential to BLB, enabling ions and metabolites to transition from the strial capillaries into the intrastrial space. Previous studies have reported “leakage” from strial capillaries after experimental interventions, inflammation, or specific genetic variants. This leakage was considered harmful to cochlear function, principally by lowering the endocochlear potential (EP). The authors of this study argue that strial capillary leakage is common across different species and conditions and does not significantly impact the EP or auditory thresholds. They provide evidence and theoretical ground that strial capillary endothelial cells and pericytes are dynamic, allowing a variable degree of permeability in response to aging, noise exposure, ototoxic drugs, autoimmune diseases, inflammation, and genetic hearing loss without adversely affecting cochlear homeostasis. Based on evidence from animal studies, the authors conclude that strial capillary leakage is not a primary driver of hearing loss and may reflect adaptive processes to different environmental conditions.

Age-related hearing loss (ARHL) is the most common sensory disorder and a significant burden to society. Despite the high prevalence of this sensory disorder, preventative and treatment strategies are lacking. Zhao and Tian reviewed the link between the Silent Information Regulator 1 (Sirtuin1 or SIRT1) and ARHL. SIRT1 plays a critical role in ARHL, tumors, and age-related neurodegenerative diseases by acting on downstream signaling pathways to mitigate DNA damage, maintain the redox balance, and reduce apoptosis. Caloric restriction, recognized as an effective measure to delay aging, activates SIRT1. This review reveals several SIRT1-activating drugs, including resveratrol and N1-methylnicotinamide, with positive anti-aging, anti-inflammatory, and antioxidant effects. It also underscores the necessity for clinical studies to establish the role of SIRT1 in the human cochlea and the timing of SIRT1 activation to prevent ARHL.

Cholesterol is an essential constituent of the cell membrane and plays a vital role in hormone synthesis, synapse formation, and cell signal transduction. Several studies have demonstrated that hypercholesterolemia is a risk factor for hearing loss. Wu et al. reviewed the impact of cholesterol homeostasis in the cochlea on the peripheral auditory function and analyzed the impact of dysregulated cholesterol homeostasis on auditory development and hereditary hearing loss. The authors analyzed changes in regulatory genes involved in cholesterol homeostasis using various hearing loss models and focused on drugs that have a therapeutic effect by regulating cholesterol homeostasis. They concluded that abnormal intracellular cholesterol homeostasis can trigger cell damage responses, such as inflammation and oxidative stress. The authors suggest that future studies should focus on the role of intracellular cholesterol in hearing maintenance and define future cholesterol-related targets for the prevention and treatment of hearing loss.

In summary, this Research Topic highlights the diverse range of approaches to SNHL. Further investigation into the underlying molecular mechanisms of SNHL and integration of novel therapy strategies into controlled clinical studies will permit significant advances in this research field and positive health outcomes.

## Author contributions

SMV: Writing – original draft, Writing – review & editing. ACV-O: Writing – review & editing. JMEC: Writing – review & editing.

